# Cofilin1-dependent actin dynamics control DRP1-mediated mitochondrial fission

**DOI:** 10.1038/cddis.2017.448

**Published:** 2017-10-05

**Authors:** Katharina Rehklau, Lena Hoffmann, Christine B Gurniak, Martin Ott, Walter Witke, Luca Scorrano, Carsten Culmsee, Marco B Rust

**Affiliations:** 1 Neurobiology/Neurophysiology Group, University of Kaiserslautern, Kaiserslautern, Germany; 2 Institute for Pharmacology and Clinical Pharmacy, University of Marburg, Marburg, Germany; 3 DFG Research Training Group ‘Membrane Plasticity in Tissue Development and Remodeling’, GRK 2213, University of Marburg, Marburg, Germany; 4 Institute of Genetics, University of Bonn, Bonn, Germany; 5 Institute for Biochemistry and Biophysics, University of Stockholm, Stockholm, Sweden; 6 Department of Biology, University of Padua, Padua, Italy; 7 Dulbecco-Telethon Institute, Venetian Institute of Molecular Medicine, Padua, Italy; 8 Molecular Neurobiology Group, Institute of Physiological Chemistry, University of Marburg, Marburg, Germany

## Abstract

Mitochondria form highly dynamic networks in which organelles constantly fuse and divide. The relevance of mitochondrial dynamics is evident from its implication in various human pathologies, including cancer or neurodegenerative, endocrine and cardiovascular diseases. Dynamin-related protein 1 (DRP1) is a key regulator of mitochondrial fission that oligomerizes at the mitochondrial outer membrane and hydrolyzes GTP to drive mitochondrial fragmentation. Previous studies demonstrated that DRP1 recruitment and mitochondrial fission is promoted by actin polymerization at the mitochondrial surface, controlled by the actin regulatory proteins inverted formin 2 (INF2) and Spire1C. These studies suggested the requirement of additional actin regulatory activities to control DRP1-mediated mitochondrial fission. Here we show that the actin-depolymerizing protein cofilin1, but not its close homolog actin-depolymerizing factor (ADF), is required to maintain mitochondrial morphology. Deletion of cofilin1 caused mitochondrial DRP1 accumulation and fragmentation, without altering mitochondrial function or other organelles’ morphology. Mitochondrial morphology in cofilin1-deficient cells was restored upon (i) re-expression of wild-type cofilin1 or a constitutively active mutant, but not of an actin-binding-deficient mutant, (ii) pharmacological destabilization of actin filaments and (iii) genetic depletion of DRP1. Our work unraveled a novel function for cofilin1-dependent actin dynamics in mitochondrial fission, and identified cofilin1 as a negative regulator of mitochondrial DRP1 activity. We conclude that cofilin1 is required for local actin dynamics at mitochondria, where it may balance INF2/Spire1C-induced actin polymerization.

Mitochondria are crucially important for a number of cellular processes including energy metabolism, Ca^2+^-buffering or apoptosis.^1, 2^ The mitochondrial network is continuously shaped by fission and fusion events, and any disturbance in mitochondrial dynamics alters mitochondrial morphology and may affect mitochondrial function.^3^ In fact, mitochondrial dynamics is crucial for the transmission of mitochondria to dividing cells, distribution of mitochondria during cellular differentiation or repair of damaged organelles.^4, 5^ Mitochondrial fusion is mediated by mitofusins and by optic atrophy 1,^6, 7^ and mitochondrial fission is mediated by dynamin-related protein 1 (DRP1).^8^ Mutations in these proteins, or their inactivation in mice, resulted in embryonic lethality or various pathologies including cancer and neurodegenerative, endocrine and cardiovascular diseases,^9, 10, 11, 12, 13, 14^ thereby highlighting the relevance of mitochondrial dynamics for the development and maintenance of the organisms.

Mitochondrial recruitment of DRP1 and its oligomerization at the outer membrane are key events in mitochondrial fission.^15^ The mechanisms that act upstream of DRP1 recruitment and oligomerization are not fully understood, but several recent studies proposed a role for actin in these processes.^16, 17, 18^ These studies evolved a model in which actin polymerization drives the initial constriction of the mitochondrial tube that is required for DRP1 oligomerization and mitochondrial fission.^16^ Moreover, they identified actin regulators relevant for mitochondrial fission, for example, inverted formin 2 (INF2) that is located in the membrane of the endoplasmic reticulum (ER) and works in conjunction with mitochondrial Spire1C.^19, 20^ Further, these studies suggested the requirement of additional actin regulatory activities to fine-tune actin dynamics at the mitochondrial surface, which may control DRP1-mediated mitochondrial fission.^16, 20^

ADF/cofilin proteins are important regulators of actin dynamics that accelerate the dissociation rate of actin subunits and sever actin filaments (F-actin).^21^ We previously reported abundant expression of two ADF/cofilin proteins in mouse embryonic fibroblasts (MEFs), namely actin-depolymerizing factor (ADF) and cofilin1.^22^ Moreover, we found that both proteins interact with mitochondria through an actin-dependent mechanism.^22^ We therefore speculated that ADF/cofilin could control actin dynamics at the mitochondrial surface and that ADF/cofilin may be relevant for DRP1-mediated mitochondrial fission. By exploiting MEFs from genetically modified mice, we here investigated whether ADF and/or cofilin1 control mitochondrial DRP1 recruitment and whether they are relevant for mitochondrial dynamics. While we found normal mitochondrial morphology in ADF-deficient MEFs, mitochondria were fragmented in cofilin1-deficient MEFs. Mitochondrial fragmentation was associated with elevated mitochondrial DRP1 levels, and mitochondrial morphology was restored by (i) re-expression either of wild-type cofilin1 or a constitutive active mutant, but not of an actin-binding-deficient mutant, (ii) pharmacological destabilization of F-actin and (iii) genetic downregulation of DRP1. In summary, we identified cofilin1-dependent actin dynamics as a novel and crucial mechanism that negatively controls DRP1-mediated mitochondrial fission.

## Results

### Mitochondrial morphology is controlled by cofilin1, but not by ADF

To study the relevance of ADF/cofilin for mitochondrial morphology in mammalian cells, we chose MEFs as a model system. Generation of MEFs deficient for either cofilin1 or ADF or both proteins from transgenic mice has been described previously.^22^ Briefly, inactivation of cofilin1 in MEFs was achieved by 4-hydroxytamoxifen (OH-TAM) treatment of floxed cofilin1 (Cfl1^flx/flx^) MEFs that stably expressed an OH-TAM-inducible variant of Cre recombinase (MCM).^23, 24^ Indeed, OH-TAM treatment (1 *μ*M) of MCM-Cfl1^flx/flx^-MEFs efficiently reduced cofilin1 levels (Supplementary Figure S1A). While residual cofilin1 expression was still present after 24 h, OH-TAM treatment for 48 h reduced cofilin1 to undetectable levels. We here performed experiments with MCM-Cfl1^flx/flx^-MEFs after 72 h of OH-TAM treatment, similar to our previous study.^22^ MEFs deficient for cofilin1 and ADF (Cfl1^−/−^/ADF^−/−^) were generated from Cfl1^flx/flx^/ADF^−/−^-MEFs by stable MCM expression and 72 h OH-TAM treatment. Cfl1^flx/flx^-MEFs treated with OH-TAM for 72 h served as controls (CTR).

**Figure 1:**
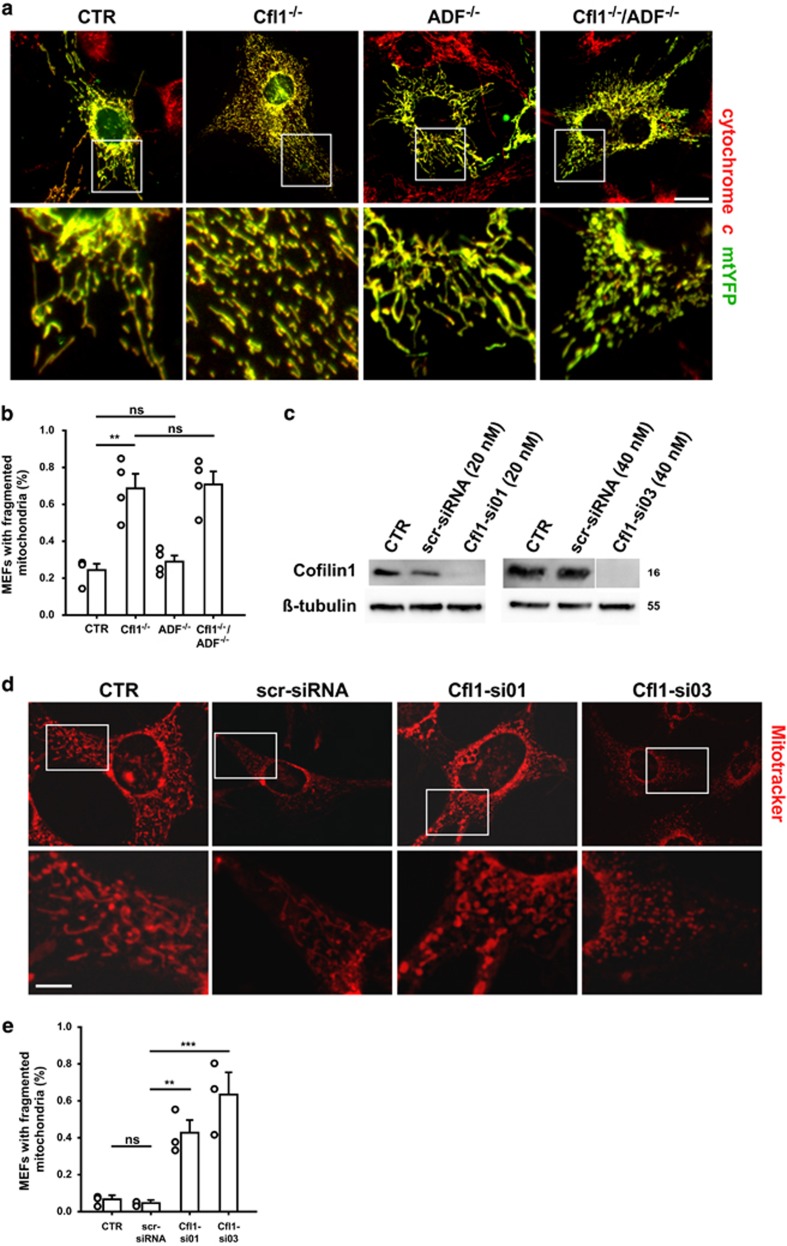
Loss of cofilin1 induced mitochondrial fragmentation in MEFs. (**a**) Shown are representative micrographs of a control MEF (CTR) and MEFs lacking either cofilin1 (Cfl1^−/−^), ADF (ADF^−/−^) or both ADF/cofilin proteins (Cfl1^−/−^/ADF^−/−^). Mitochondria visualized by mitochondrial-targeted YFP (mtYFP, green) and cytochrome *c* immunoreactivity (red) appeared fragmented in Cfl1^−/−^- and Cfl1^−/−^/ADF^−/−^-MEFs, but not in ADF^−/−^-MEFs. White boxes indicate areas shown in high magnification. Scale bars: 50 *μ*m. (**b**) Relative MEF number with fragmented mitochondria was increased in Cfl1^−/−^- and Cfl1^−/−^/ADF^−/−^-MEFs, but not in ADF^−/−^-MEFs (CTR: 24.4±3.4%, *n*=214 cells/4 independent experiments; ADF^−/−^: 28.9±3.3%, *n*=149/4, *P*=0.376; Cfl1^−/−^: 68.6±8.0%, *n*=241/4, *P*<0.01; Cfl1^−/−^/ADF^−/−^: 70.8±7.0%, *n*=184/4, *P*<0.01). (**c**) Western blots demonstrating reduced cofilin1 levels upon transfection with either Cfl1-si01 or Cfl1-si03 in CTR-MEFs. Transfection of a control siRNA (scr-siRNA) did not change cofilin1 levels. *β*-tubulin served as a loading control. (**d**) Representative micrographs of Mitotracker-stained CTR-MEFs. Fragmented mitochondria were noted upon transfection of either Cfl1-si01 or Cfl1-si03, but not of scr-siRNA. White boxes indicate areas shown in high magnification. Scale bars: 50 *μ*m. (**e**) Relative MEF numbers with fragmented mitochondria were increased upon transfection of either Cfl1-si01 or Cfl1-si03, but not of scr-siRNA (CTR: 6.0±1.6% scr-siRNA: 4.0±0.7% Cfl1-si01: 42.1±6.7%, *P*<0.01; Cfl1-si03: 62.8±11.3%, *P*<0.001; *n*>900 MEFs in three independent experiments for each condition). Columns and error bars in (**b**), F: mean values (MV) and standard error of the mean (S.E.M.). Open circles: values of independent experiments. **P<0.01, NS: not significant

We visualized mitochondria in mutant MEFs by cytochrome *c* immunoreactivity or by the expression of a yellow fluorescent protein containing a mitochondrial targeting sequence (mtYFP) (Figure 1a). Mitochondria appeared normal in ADF^−/−^-MEFs. Instead, they were clearly fragmented in MEFs lacking either cofilin1 alone or both ADF/cofilin proteins. Time course of mitochondrial fragmentation in OH-TAM-treated, MCM-Cfl1^flx/flx^-MEFs paralleled depletion of cofilin1 (Supplementary Figures S1B and C). Importantly, we noted no changes in mitochondrial morphology in OH-TAM-treated Cfl1^flx/flx^-MEFs (Supplementary Figures S1B and C), thereby demonstrating that mitochondrial fragmentation is a consequence of cofilin1 inactivation, but not of OH-TAM treatment. To quantify mitochondrial fragmentation in mutant MEFs, we determined the relative number of MEFs with ≥50% of mitochondrial particles possessing a longitudinal axis shorter than 1 *μ*m, similar to previous studies.^7, 25^ While this number was unchanged in ADF^−/−^-MEFs, it was almost three-fold increased in Cfl1^−/−^- or Cfl1^−/−^/ADF^−/−^-MEFs (Figure 1b). Such a procedure is adequate for determining mitochondrial fragmentation as our detailed morphometric analysis of randomly chosen MEFs revealed a 45% reduction in mitochondrial organelle size in Cfl1^−/−^-MEFs (Supplementary Figure S1D), that was associated with a 62% increase in the number of mitochondrial particles (Supplementary Figure S1E). Moreover, mitochondria organelles were rounder and less branched in Cfl1^−/−^-MEFs as deduced from a reduction in the form factor (Supplementary Figure S1F), a parameter of particle shape that has been used before to determine mitochondrial morphology.^26, 27, 28^

To prove the relevance of cofilin1 for mitochondrial morphology in an independent approach, we genetically depleted cofilin1 in CTR-MEFs by exploiting two different siRNAs (Cfl1-si01, Cfl1-si03). Both Cfl1-siRNAs efficiently reduced cofilin1 mRNA levels (data not shown), and cofilin1 protein levels were reduced below detection limits (Figure 1c). A scrambled siRNA (scr-siRNA) was used as a control, and it did not change cofilin1 levels. Mitochondria appeared fragmented in CTR-MEFs upon transfection of either Cfl1-si01 or Cfl1-si03, but not of scr-siRNA (Figure 1d). Indeed, careful quantification revealed an increased MEF number with fragmented mitochondria upon transfection of either Cfl1-si01 or Cfl1-si03, while this number was unchanged upon transfection of scr-siRNA (Figure 1e).

Together, our data demonstrated mitochondrial fragmentation upon genetic inactivation of cofilin1 either by OH-TAM treatment of MCM-Cfl1^flx/flx^-MEFs or by siRNA-mediated knockdown in CTR-MEFs. Lack of mitochondrial fragmentation in MCM-Cfl1^flx/flx^-MEFs upon 24 h OH-TAM treatment suggested that residual cofilin1 was sufficient to maintain mitochondrial morphology. Mitochondrial morphology was unchanged in ADF^−/−^ MEFs, and mitochondrial fragmentation in Cfl1^−/−^/ADF^−/−^-MEFs was similar to Cfl1^−/−^-MEFs. Hence, we identified cofilin1 as a crucial regulator of mitochondrial morphology, while its close homolog ADF was dispensable for mitochondrial morphology.

### Cofilin1 inactivation specifically impairs mitochondrial morphology

We next tested whether mitochondrial fragmentation in Cfl1^−/−^-MEFs was associated with defects in mitochondrial function or cellular energy metabolism. Interestingly, we found no changes in mitochondrial membrane potential or mitochondrial production of reactive oxygen species (ROS) in Cfl1^−/−^-MEFs (Supplementary Figures S2A–D). Moreover, ATP production and the mitochondrial oxygen consumption rate (OCR) was unchanged in mutant MEFs (Supplementary Figures S2E and F). Additionally, glycolysis as deduced from the extracellular acidification rate (ECAR) was unchanged in Cfl1^−/−^-MEFs (Supplementary Figure S2G). Hence, fragmentation of mitochondria in Cfl1^−/−^-MEFs was not associated with any defects in mitochondrial function or cellular energy metabolism. Results were very similar in non-treated MCM- Cfl1^flx/flx^-MEFs that we used as additional controls, thereby excluding any adverse effect of OH-TAM on mitochondrial function (data not shown).

Interestingly, antibody staining against the ER retention sequence KDEL, protein disulfide isomerase (PDI), 130 kDa *cis*-Golgi matrix protein (GM130), giantin and *β*-tubulin revealed the absence of any obvious morphological changes of the ER, the Golgi apparatus and the microtubule cytoskeleton in Cfl1^−/−^-MEFs (Supplementary Figure S3). Hence, while inactivation of cofilin1 markedly altered mitochondrial morphology, it did not induce any obvious structural defects in cell organelles that intimately interact with mitochondria. Together, our data suggested a specific function for cofilin1 in mitochondrial morphology.

### CofiIin1-mediated actin dynamics controls mitochondrial morphology

**Figure 2:**
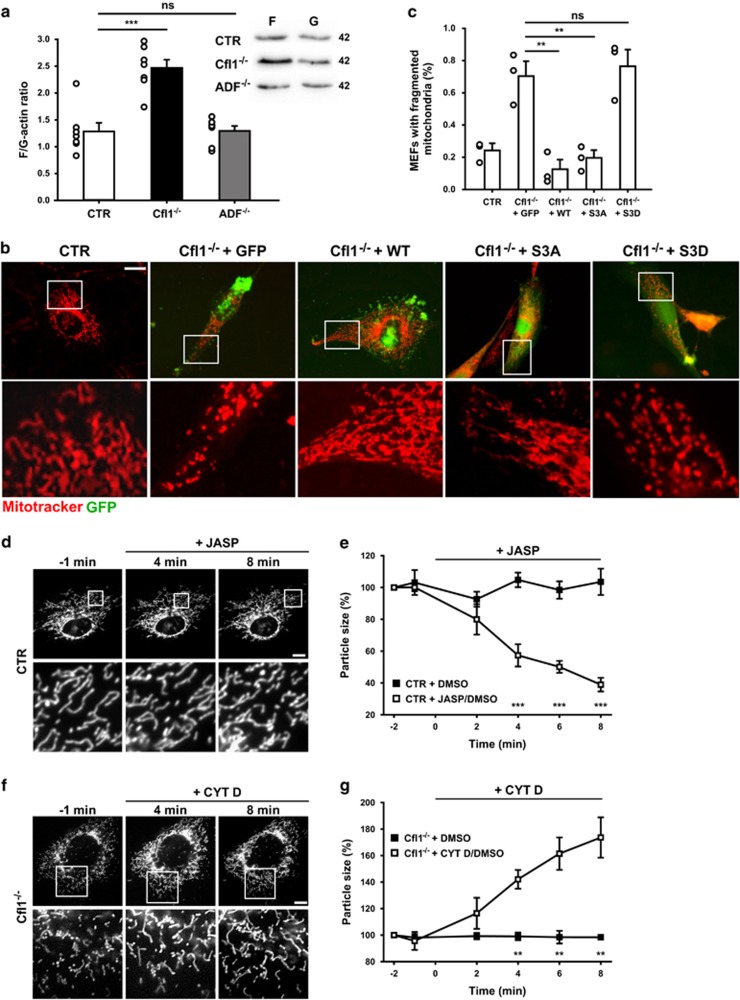
Mitochondrial fragmentation in Cfl1^−/−^ MEFs was caused by impaired actin dynamics. (**a**) Representative western blots showing actin levels in insoluble (f) and soluble (g) protein fractions from CTR-, Cfl1^−/−^- and ADF^−/−^-MEFs. Actin levels in both protein fractions were quantified to calculate the F/G-actin ratio. Compared with CTR-MEFs, the F/G-actin ratio was strongly increased in Cfl1^−/−^-MEFs, but not in ADF^−/−^-MEFs (CTR: 1.28±0.16; Cfl1^−/−^: 2.46±0.16, *P*<0.001; ADF^−/−^: 1.29±0.09, *P*=0.959; *n*=7 for each group). (**b**) Representative micrographs of Mitotracker-stained Cfl1^−/−^-MEFs upon expression of either GFP or various GFP-tagged cofilin1 variants: WT-Cfl1, constitutive active Cfl1-S3A, or constitutive inactive Cfl1-S3D. (**c**) Quantification of relative MEF numbers with fragmented mitochondria revealed that expression of either WT-Cfl1 or Cfl1-S3A, but not of GFP or Cfl1-S3D restored mitochondrial morphology in Cfl1^−/−^-MEFs (Cfl1^−/−^+GFP: 69.9±9.1; Cfl1^−/−^+WT-Cfl1: 12.1±5.7, *P*<0.01; Cfl1^−/−^+Cfl1-S3A: 19.1±4.4, *P*<0.01; Cfl1^−/−^+Cfl1-S3D: 76.1±10.4; *n*>850/3 for each condition). (**d**) Representative micrographs of a CTR-MEF before (−1 min) and after (+4 min, +8 min) treatment with the F-actin stabilizing drug jasplakinolide (JASP) that was added at time point 0 min. (**e**) Graph showing JASP-induced mitochondrial fragmentation in CTR-MEFs. For example, upon 4 min of JASP treatment, mitochondrial size was clearly reduced (57.3±7.0%, *P*<0.001, *n*=5 MEFs in five independent experiments) and it reduced to roughly 40% of basal levels after 8 min (38.9±4.3%, *P*<0.001). Conversely, dimethyl sulfoxide (DMSO) did not change mitochondrial morphology (8 min: 103.6±8.3%, *n*=5/5). (**f**) Representative micrographs of a Cfl1^−/−^-MEF before (−1 min) and after (+4 min, +8 min) treatment with the F-actin destabilizing drug cytochalasin D (CYTD) that was added at time point 0 min. (**g**) Graph showing CYTD-induced mitochondrial elongation in Cfl1^−/−^-MEFs (4 min: 142.1±7.1%, *P*<0.01, *n*=5/5; 8 min: 173.7±15.2%, *P*<0.01). Conversely, DMSO did not change mitochondrial morphology (8 min: 98.4±1.7%, *n*=4/4). White boxes in (**b**), (**d**) and (**f**) indicate areas shown in high magnification. Scale bars in (**b**), (**d**) and (**f**): 50 *μ*m. Columns and error bars in (**a**) and (**c**): MV+S.E.M. Open circles: values of independent experiments. Squares in (**e**) and (**g**): MV+S.E.M. **P<0.01; ***P<0.001; NS: not significant

Cofilin1 plays a key role in actin dynamics as it can sever F-actin and increase the dissociation rate of actin subunits.^21^ We therefore expected elevated F-actin levels in Cfl1^−/−^-MEFs and a shift in the ratio of F-actin to monomeric, globular (G) actin (F/G-actin ratio). To test this, we separated the insoluble protein fraction including F-actin from the soluble protein fraction containing G-actin and resolved both protein fractions in equal amounts of F-actin stabilizing PHEM buffer. Thereafter, equal volumes of both fractions were loaded on a gel, and absolute actin levels were quantified in both fractions by western blotting to calculate the F/G-actin ratio, similar to previous studies.^29, 30^ Compared with CTR-MEFs, the F/G-actin ratio was almost doubled in Cfl1^−/−^-MEFs, but unchanged in ADF^−/−^-MEFs (Figure 2a). Hence, mitochondrial fragmentation in Cfl1^−/−^-MEFs was associated with a disequilibrium of F- to G-actin, while ADF inactivation changed neither mitochondrial morphology nor the F/G-actin ratio.

Cofilin1’s function in actin dynamics is controlled by de-/phosphorylation of a conserved serine residue at position 3 (S3).^21, 31^ S3 dephosphorylation enables cofilin1 to interact with actin, while S3 phosphorylation inhibits actin binding. If cofilin1 controls mitochondrial morphology via an actin-dependent mechanism, mitochondrial morphology in Cfl1^−/−^-MEFs should be restored by the expression of a ‘non-phosphorylatable’ (active) cofilin1 mutant, but not by a phospho-mimetic (inactive) cofilin1 mutant. To test this, we re-expressed various GFP-tagged cofilin1 variants in Cfl1^−/−^-MEFs: wild-type cofilin1 (WT-Cfl1), a constitutive active mutant with alanine at position 3 (Cfl1-S3A) and a constitutive inactive mutant with aspartate at position 3 (Cfl1-S3D). Such mutants have been used before to study cofilin1-dependent actin dynamics.^32, 33, 34, 35, 36^ We compared mitochondrial fragmentation in Cfl1^−/−^-MEFs expressing these cofilin1 variants to Cfl1^−/−^-MEFs transfected with a GFP control plasmid. As expected, expression of GFP did not change mitochondrial morphology in Cfl1^−/−^-MEFs (Figure 2b), and the relative number of GFP-expressing Cfl1^−/−^-MEFs with fragmented mitochondria was higher when compared with CTR-MEFs (Figure 2c). Instead, expression of WT-Cfl1 restored mitochondrial morphology in Cfl1^−/−^-MEFs, and the relative MEF number with fragmented mitochondria was reduced when compared with GFP-expressing Cfl1^−/−^-MEFs. Similarly, expression of the constitutive active mutant Cfl1-S3A restored mitochondrial morphology in Cfl1^−/−^-MEFs, while mitochondria remained fragmented in Cfl1^−/−^-MEFs that expressed Cfl1-S3D. Together, our data suggested that cofilin1 controlled mitochondrial morphology via an actin-dependent mechanism.

Our results (increased F-actin levels, rescue of mitochondrial morphology upon expression of Cfl1-S3A, but not of Cfl1-S3D) let us hypothesize that cofilin1 controls actin dynamics at the mitochondrial surface and that F-actin stabilization in the absence of cofilin1 caused mitochondrial fragmentation. If our hypotheses were true, pharmacological stabilization of F-actin, for example, by the actin stabilizing drug jasplakinolide (JASP),^37^ should induce mitochondrial fragmentation in CTR-MEFs, similar to the changes we observed in Cfl1^−/−^-MEFs. To test this, we performed live cell microscopy on mtRFP-expressing CTR-MEFs and we determined relative changes in mitochondrial organelle size upon JASP treatment as an indicator for mitochondrial fragmentation (Figure 2d). Indeed, mitochondrial size was reduced as early as 4 min upon JASP treatment (Figure 2e), and it decreased to roughly 40% of basal levels after 8 min. Conversely, no changes in organelle size were noted in dimethyl sulfoxide (DMSO) control experiments. Hence, JASP-induced stabilization of F-actin resulted in mitochondrial fragmentation in CTR-MEFs, similar to genetic depletion of cofilin1.

To prove whether mitochondrial fragmentation in Cfl1^−/−^-MEFs was caused by increased F-actin levels, we again performed time lapse experiments and quantified mitochondrial size in mtRFP-expressing Cfl1^−/−^-MEFs before and after F-actin destabilization induced by cytochalasin D (CYTD), a potent inhibitor of actin polymerization.^38^ Indeed, CYTD treatment significantly increased mitochondrial size in Cfl1^−/−^-MEFs (Figures 2f and g), while DMSO did not affect mitochondrial size. Thus, mitochondrial morphology in Cfl1^−/−^-MEFs was restored by drug-induced F-actin destabilization, confirming that mitochondrial fragmentation in Cfl1^−/−^-MEFs was caused by defective actin dynamics.

### F-actin stabilization correlates with increased mitochondrial DRP1 levels

**Figure 3:**
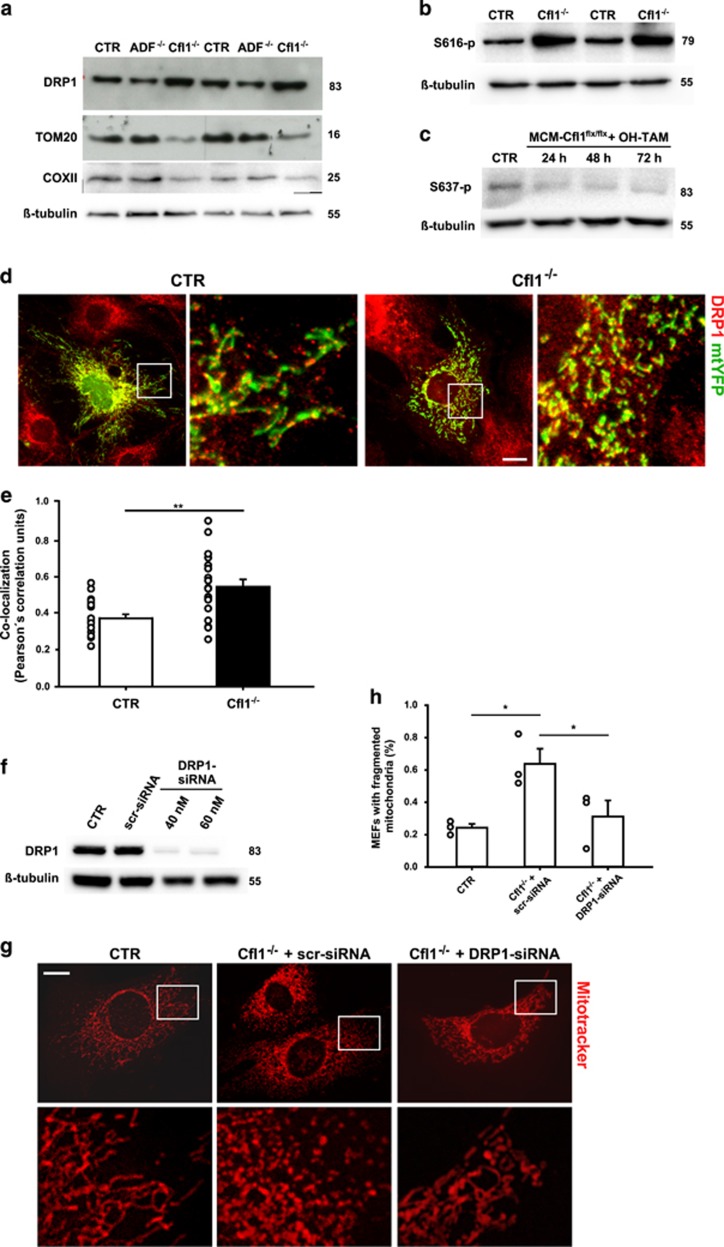
Mitochondrial fragmentation in Cfl1^−/−^ MEFs was mediated by DRP1. (**a**) Western blot of two independent experiments demonstrating increased DRP1 levels in Cfl1^−/−^-MEFs, while TOM20 and COXII expression levels were reduced. No changes in DRP1, TOM20 or COXII expression was noted in ADF^−/−^-MEFs. (**b**) Western blot of two independent experiments demonstrating increased phosphorylation of DRP1 at S616 in Cfl1^−/−^-MEFs. (**c**) Representative western blot demonstrating reduced phosphorylation of DRP1 at S637 in Cfl1^flx/flx^-MEFs upon OH-TAM treatment. *β*-Tubulin was used as a loading control in (**a**–**c**). (**d**) DRP1 immunoreactivity (red) in representative mtYFP-expressing (green) CTR- and Cfl1^−/−^-MEFs. (**e**) In Cfl1^−/−^-MEF, the Pearson's correlation of mtYFP and DRP1 was increased (CTR: 0.36±0.02; KO: 0.54±0.04; *n*=19, *P*<0.01). (**f**) siRNA against DRP1 efficiently depleted DRP1 in MEFs, while a scrambled control siRNA (scr-siRNA) did not alter DRP1 levels. *β*-Tubulin was used as a loading control. (**g**) Representative micrographs of a Mitotracker-stained CTR-MEF and of Cfl1^−/−^-MEFs upon transfection with either scr-siRNA or DRP1-siRNA. Mitochondria in Cfl1^−/−^-MEFs appeared fragmented upon transfection with scr-siRNA. Conversely, transfection of DRP1-siRNA (40 nM) restored mitochondrial morphology in Cfl1^−/−^-MEFs. (**h**) Quantification of relative MEF numbers with fragmented mitochondria revealed that DRP1-siRNA, but not scr-siRNA, restored mitochondrial morphology in Cfl1^−/−^ MEFs (CTR: 23.2±2.7% Cfl1^−/−^+scr-siRNA: 59.8±11.5%, *P*<0.05; Cfl1^−/−^+DRP1-siRNA: 30.7±10.5, *P*<0.05; *n*>900/3 for each condition). Columns and error bars in (**e**) and (**h**): MV+S.E.M. Open circles: values of independent experiments. **P<0.01. White boxes in (**d**) and (**g**) indicate areas shown in high magnification. Scale bar in (**d**) and (**g**): 50 *μ*m

Mitochondrial fragmentation can result from increased mitochondrial fission, and DRP1 emerged as a key regulator of this process.^39^ Recent studies identified actin polymerization at the mitochondrial surface as a positive regulator of DRP1-mediated mitochondrial fission.^19, 20^ We therefore hypothesized that DRP1 is involved in mitochondrial fission in Cfl1^−/−^-MEFs. To test this, we first determined total DRP1 protein levels and found it increased by roughly 20% in two independent experiments in Cfl1^−/−^-MEFs (Figure 3a). Increased DRP1 protein levels in Cfl1^−/−^-MEFs were associated with a transient increase in DRP1 mRNA levels that reached statistical significance upon 24 h of OH-TAM treatment, but not at later time points (Supplementary Figure S4A). In contrast to DRP1, protein levels of mitochondrial markers such as TOM20 or COX2 were strongly reduced in Cfl1^−/−^-MEFs by roughly 70% and 60%, respectively (Figure 3a), suggesting an overall reduction in mitochondrial content. Unlike Cfl1^−/−^-MEFs, protein levels of DRP1, TOM20 or COXII were unchanged in ADF^−/−^-MEFs (Figure 3a).

Mitochondrial activity of DRP1 is controlled by post-translational modifications, and phosphorylation at S616 promotes mitochondrial recruitment of DRP1 and mitochondrial fission,^40, 41^ while phosphorylation at S637 inhibits both processes.^36, 42^ We next tested whether mitochondrial fragmentation in Cfl1^−/−^-MEFs was associated with altered S616 and/or S637 phosphorylation. Indeed, we found strongly increased S616 phosphorylation in Cfl1^−/−^-MEFs (Figure 3b), while S637 phosphorylation was reduced by roughly 50% of control levels 48 or 72 h upon OH-TAM treatment (Figure 3c). Together, these data suggested elevated mitochondrial recruitment of DRP1. Increased mitochondrial DRP1 localization was confirmed by immunocytochemistry (Figure 3d). Compared with CTR-MEFs, the Pearson's correlation coefficient of mtYFP and DRP1 was increased by 50% in Cfl1^−/−^-MEFs (Figure 3e). Interestingly, JASP-induced F-actin stabilization did not change total DRP1 levels in CTR-MEFs, but it increased S616 phosphorylation (Supplementary Figure S4B). Moreover, JASP treatment increased mitochondrial localization in CTR-MEFs, as deduced from increased DRP1-mtYFP co-localization (Supplementary Figures S4C and D). Together, our data revealed elevated mitochondrial DRP1 levels upon F-actin stabilization induced either by genetic inactivation of cofilin1 or by pharmacological treatment.

### DRP1 inactivation rescues mitochondrial morphology in cofilin1-deficient MEFs

Our data let us hypothesize a role for DRP1 in mitochondrial fragmentation in cofilin1-deficient MEFs. To test this hypothesis, we determined mitochondrial morphology in Cfl1^−/−^-MEFs upon siRNA-mediated genetic depletion of DRP1. Control experiments revealed efficient knockdown of DRP1 by siRNA, while a scrambled control siRNA did not affect DRP1 levels (Figure 3f). Mitochondria were fragmented in scr-siRNA-treated Cfl1^−/−^-MEFs, but they appeared less fragmented in mutant MEFs upon DRP1 knockdown (Figure 3g). Indeed, compared with CTR-MEFs, the number of fragmented mitochondria was increased in scr-siRNA-treated Cfl1^−/−^-MEFs (Figure 3h), and DRP1-siRNA halved the number of mutant MEFs with fragmented mitochondria when compared with scr-siRNA. These data revealed that DRP1 acts downstream of cofilin1 in mitochondrial morphology and that mitochondrial fragmentation in Cfl1^−/−^-MEFs was caused by enhanced mitochondrial DRP1 activity.

## Discussion

In the present study, we report mitochondrial fragmentation upon genetic inactivation of the actin-depolymerizing protein cofilin1. In Cfl1^−/−^-MEFs, mitochondrial fragmentation was associated with elevated mitochondrial DRP1 levels, and mitochondrial morphology was restored (i) by a constitutive active cofilin1 mutant, but not by an actin-binding-deficient cofilin1 mutant, (ii) by acute destabilization of F-actin and (iii) by siRNA-mediated DRP1 knockdown. Hence, our data identified cofilin1-dependent actin dynamics as a crucial negative regulator of mitochondrial DRP1 activity, thereby controlling mitochondrial fission.

While we found fragmented mitochondria upon genetic inactivation of cofilin1, mitochondrial morphology was unchanged in ADF-deficient MEFs. This was an unexpected finding because (i) cofilin1 and ADF are both abundant in MEFs,^22^ (ii) cofilin1 and ADF can both interact with mitochondria^22, 33, 43, 44^ and (iii) both proteins share very similar functions in actin dynamics that may differ quantitatively.^31^ In Cfl1^−/−^-MEFs, mitochondrial fragmentation was associated with an increased F/G-actin ratio, while ADF^−/−^-MEFs displayed no changes in mitochondrial morphology or F/G-actin ratio. These data let us conclude that cofilin1 is the major actin-depolymerizing protein in MEFs and in mitochondrial morphology. Notably, mitochondrial fragmentation in double mutant MEFs lacking cofilin1 and ADF was similar to that in Cfl1^−/−^-MEFs, and we therefore concluded that ADF is dispensable for mitochondrial morphology. Hence, while overlapping and redundant functions have been described for cofilin1 and ADF in vesicle exocytosis, neuritogenesis, turnover of contractile actin stress fibers, myelination, platelet formation or behavior,^30, 45, 46, 47, 48, 49^ only cofilin1 is relevant for mitochondrial morphology. Different upstream regulatory mechanism have been identified for cofilin1 and ADF,^31^ which may be relevant in the context of mitochondrial dynamics.

Notably, mitochondrial fragmentation was not associated with any obvious defects in mitochondrial function or cellular energy metabolism, as we found no changes in mitochondrial ROS and ATP production, OCRs, glycolysis or mitochondrial membrane potential in Cfl1^−/−^-MEFs. Instead, the reduction of mitochondrial content in Cfl1^−/−^-MEFs that we deduced from reduced TOM20 and COXII expression levels rather suggested slightly improved mitochondrial functions. Absence of mitochondrial dysfunction in Cfl1^−/−^-MEFs was also evident from our previous study in which we showed normal sensitivity to staurosporine- or H_2_O_2_-induced cytochrome *c* release and apoptotic cell death.^22^ In Cfl1^−/−^-MEFs, we found no obvious changes in the morphology of the ER, the Golgi apparatus or the microtubule cytoskeleton. These data excluded severe structural defects in Cfl1^−/−^-MEFs and indicated that the observed mitochondrial fragmentation did not occur secondary to other structural changes, for example, of the ER or the microtubule cytoskeleton that both intimately interact with mitochondria and that reportedly influence mitochondrial morphology in mammalian cells.^19, 50, 51^ Additionally, from our previous study, we excluded that mitochondrial fragmentation in Cfl1^−/−^-MEFs was a consequence of an increased apoptotic index.^22^ Together, our data indicated a direct and specific function for cofilin1 in mitochondrial morphology, without immediate functional consequences for mitochondria. We therefore propose that cofilin1 controls mitochondrial dynamics in a physiological manner, which has been postulated as a prerequisite for physiological development and function, for example, mitochondrial fragmentation and concomitant maintenance of mitochondrial function is required for mitochondrial quality control, repair mechanisms or transport to cellular compartments with a high energy demand.^15, 52, 53, 54^ Cofilin1-dependent mitochondrial fission may thus support the organelles’ dynamics required for cellular function and maintenance, and this physiological mitochondrial division is in sharp contrast to pathological conditions of cellular stress, where DRP1-dependent fission was accompanied by loss of mitochondrial integrity and function, release of apoptotic factors and accumulation around the nucleus.^55, 56, 57, 58^ In MEFs, inactivation of cofilin1 did not affect apoptosis signaling,^22^ and further studies are required to clarify the different modes of mitochondrial fission in development, under physiological conditions and in cell death and disease.

A function of cofilin1 as a negative regulator of mitochondrial fragmentation is in good agreement with a previous study in which mitochondria appeared elongated in COS-7 cells upon mitochondrial targeting of cofilin1.^43^ Moreover, cofilin1 has been implicated in mitochondrial dynamics downstream of the transcription factor serum response factor (SRF).^34^ Specifically, this study reported (i) a rescue of fragmented mitochondria in SRF-deficient neurons upon overexpression either of constitutive active cofilin1 or of the cofilin1-activating phosphatase slingshot and (ii) that overexpression of constitutive inactive cofilin1 induced mitochondrial fragmentation in neurons. Together, these findings prove that cofilin1’s function as a negative regulator of mitochondrial fragmentation is not restricted to MEFs. Moreover, they let to the suggestion that mitochondrial cofilin1 exerts a protective function and that any dysregulation may contribute to the pathology of, for example, neurodegenerative diseases,^34^ as it has been discussed for Alzheimer's disease.^59, 60^

Opposite to the proposed function of cofilin1 as a negative regulator of mitochondrial fragmentation, recent findings in mammalian breast cancer cells suggested that cofilin1 promoted mitochondrial fission in tumor cells.^36^ In that study, knockdown of cofilin1 efficiently blocked mitochondrial fragmentation induced by the natural compound erucin. However, erucin not only induced mitochondrial fragmentation, but also apoptosis, and cofilin1 inactivation efficiently blocked erucin-induced apoptosis in this model system.^36^ An important role for cofilin1 in apoptosis has been shown in several recent studies that mainly focused on signaling cascades upstream of cofilin1.^33, 36, 43, 61, 62^ Another study, however, linked cofilin1 directly to the opening of the mitochondrial permeability transition pore.^33^ Via this pathway, cofilin1 can promote cytochrome *c* release and apoptosis progression, a function that is independent of its role in mitochondrial dynamics. Hence, cofilin1-dependent regulation of mitochondrial morphology in paradigms of apoptosis may rather be indirect, depending primarily on its contribution to mitochondrial damage as a crucial step during apoptosis execution. We previously showed normal apoptosis progression in cofilin1-deficient MEFs,^22^ thereby suggesting cell-type specific functions for cofilin1 in apoptosis.^22, 61, 63, 64, 65^ Hence, cofilin1-deficient MEFs allowed us to dissect the function of cofilin1 in mitochondrial morphology, independent of the detrimental functional and morphological changes of mitochondria that accompany apoptosis.

**Figure 4:**
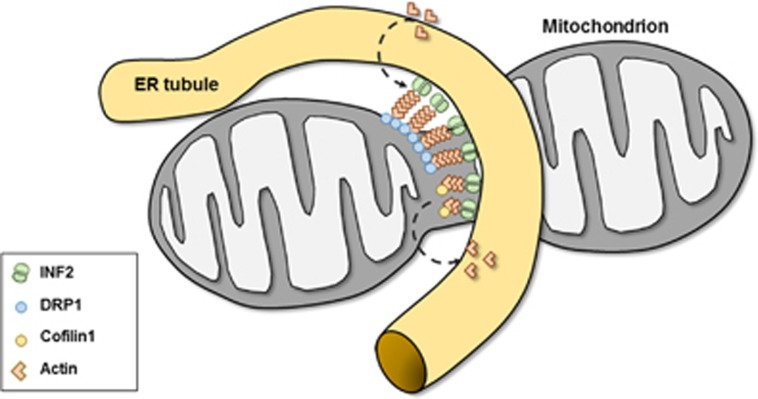
Putative model for the role of cofilin1 in mitochondrial morphology**.** Previous studies revealed that actin polymerization induced by ER-anchored INF2 is relevant for mitochondrial DRP1 oligomerization and mitochondrial fission and that INF2 cooperates with mitochondrial Spire1C (not shown).^19, 20^ We found fragmented mitochondria and elevated mitochondrial levels of DRP1 in cofilin1-deficient MEFs. Further, we found restored mitochondrial morphology in cofilin1-deficient MEFs (i) upon expression of a constitutive active cofilin1 mutant, but not upon expression of a cofilin1 mutant that does not bind actin, (ii) upon acute F-actin destabilization (iii) and upon genetic inhibition of DRP1. Our data promote a model in which cofilin1-dependent actin dynamics acts as a negative regulator of mitochondrial DRP1 activity and mitochondrial fission. Cofilin1-dependent actin depolymerization might be required for fine-tuning actin dynamics at the mitochondrial surface by antagonizing INF2/Spire1C-mediated actin polymerization

Different experimental conditions (physiological mitochondrial fission *versus* apoptotic mitochondrial fragmentation) may explain the discrepancies between our data and those on cofilin1 in erucin-induced apoptosis.^36^ However, a recent study by Li *et al.*^44^ suggested that cofilin1 worked in conjunction with cortactin and the Arp2/3 complex to promote mitochondrial fission. These authors demonstrated mitochondrial elongation in MEFs upon siRNA-mediated cofilin1 knockdown that was associated with a mitochondrial accumulation of DRP1, and they concluded that mitochondrial DRP1 was inactive in cofilin1-depleted MEFs and that additional stimuli were required to complete mitochondrial fission.^44^ In contrast, here we found fragmented mitochondria upon genetic inactivation of cofilin1 using two independent approaches: Cre-mediated cofilin1 knockout in MCM-Cfl1^flx/flx^-MEFs and siRNA-mediated cofilin1 gene silencing in CTR-MEFs. In cofilin1-depleted MEFs, mitochondrial fragmentation was associated with a mitochondrial accumulation of DRP1 and elevated mitochondrial DRP1 activity, and we therefore suggested that cofilin1 controls mitochondrial morphology via negatively regulating mitochondrial DRP1 activity. Since we could rescue mitochondrial fragmentation in mutant MEFs by acute F-actin destabilization and by the expression of a constitutive active mutant, but not by an actin-binding-deficient mutant, we hypothesized that cofilin1 controls mitochondrial DRP1 activity via an actin-dependent mechanism. Such a scenario is in good agreement with several recent studies that identified actin polymerization at the mitochondrial surface as a crucial determinant of mitochondrial DRP1 recruitment and fission.^16, 17, 18, 19, 20, 44^ If our hypothesis holds true, mitochondrial morphology in Cfl1^−/−^-MEFs should be restored by DRP1 inactivation. Indeed, we found normal mitochondrial morphology in Cfl1^−/−^-MEFs upon DRP1 silencing. Hence, we identified cofilin1-dependent actin dynamics as a crucial regulator of mitochondrial morphology that inhibits mitochondrial transactivation of DRP1 and, thereby, mitochondrial fission. We propose that cofilin1 is required to fine-tune actin dynamics at the mitochondrial surface, where it may counteract INF2/Spire1C-mediated actin polymerization, which previously has been shown to promote mitochondrial fission (Figure 4).

## Materials and methods

### Cell culture conditions

MEFs were cultured in Dulbecco’s modified Eagle’s medium (DMEM; Invitrogen, Carlsbad, CA, USA), supplemented with 10% (v/v) fetal calf serum (PAA), penicillin (100 units/ml, PAA) and streptomycin (100 *μ*g/ml, PAA) at 37 °C and 5% CO_2_ atmosphere in a humid cell incubator (Jouan IG-150; Jouan, Saint-Herblain, France).

### Transient transfection

MEFs were cultured in antibiotic-free culture medium at a density of 80 000 cells/ml on glass slides (diameter: 40 mm) coated with poly-l-lysine (Invitrogen). 18–24 h later, MEFs were transfected by using either TransFectin Lipid Reagent (Biorad, Hercules, CA, USA) or FuGENE HD Transfection Reagent (Promega, Madison, WI, USA) according to the manufacturer's protocols. Medium was replaced with regular culture medium 4–6 h after transfection, and MEFs were used for experiments 24 h after transfection. Generation of constructs has been described previously: mtYFP and mtRFP.^66^

### Immunocytochemistry

MEFs were fixed in 4% paraformaldehyde (PFA) in phosphate-buffered saline (PBS; containing (in mM) 2.7 KCl, 1.5 KH_2_PO_4_, 137 NaCl, 8.1 Na_2_HPO_4_; pH 7.4) for 15 min at 4 °C, washed twice in PBS and stored at 4 °C in PBS containing 0.1% NaN_3_ until further processing. Permeabilization of MEFs were obtained by 1 h incubation at room temperature (RT) in blocking solution containing 0.3% Triton X-100 (Fluka, Buchs, Switzerland), 10 mM 4-(2-hydroxyethyl)-1-piperazineethanesulfonic acid (HEPES, pH 7.4, Sigma Aldrich, St. Loius, MO, USA) and 3% bovine serum albumin (AppliChem, Darmstadt, Germany). Thereafter, MEFs were incubated with primary antibodies either for 2 h at RT or overnight (o/n) at 4 °C, incubation time and temperature as well as dilution of primary antibodies is indicated below. After that, MEFs were washed twice in PBS and incubated for 1 h at RT with the appropriate secondary antibody (Alexa Fluor 488- or Alexa Fluor 546-conjugated goat anti-mouse IgG or goat anti-rabbit IgG; Invitrogen) diluted 1:1000 in blocking solution. To determine co-localization of proteins, we calculated the Pearson's correlation by using ImageJ plugin 'just another co-localization plugin' (National Institutes of Health) as described before.^67^

### Live cell imaging

MEFs seeded at a density of 40 000 cells per ml onto 35 mm-FluoroDishes (World Precision Instruments) were transfected with either mtRFP or mtYFP. Twenty-four hours after transfection, MEFs were supplemented with 10 mM HEPES and transferred to an Axio Observer Z1 microscope (Carl Zeiss, Oberkochen, Germany), the incubation chamber was pre-heated to 37 °C. For each experiment, images were captured at 10 s intervals using an Axio Cam MR3 (Carl Zeiss). In some experiments, actin drugs (0.25 *μ*m JASP or 0.5 *μ*M CYTD, both diluted in DMSO) were directly applied to the culture medium 10 min after starting the recordings, indicated by time point 0 min on the abscissae of Figures 2e and g.

### Mitochondrial morphology

Mitochondrial morphology for Figure 1a was analyzed in MEFs expressing either mtRFP or mtYFP. For each experiment, at least 20 randomly chosen cells were taken at an inverted Nikon A1R confocal microscope (Nikon, Minato, Japan) using a 40xCFI Plan Fluor 1.3 objective. Experiments were repeated at least three times (≥4 independent experiments). Images were processed and analyzed by using ImageJ software (version 1.45 h; NIH, Rockville, MD, USA). Background signal was reduced with the rolling ball (radius: 20 pixels) background subtractions tool. Thereafter, fluorescent images were converted into binary images using the automated threshold function. To quantify mitochondrial fragmentation, MEFs were categorized into the groups 'cells with predominantly fragmented mitochondria' and 'cells with predominantly tubular mitochondria', similar to previous studies.^7, 25^ Briefly, the length of the major axis of an ellipse equivalent to each individual mitochondrial particle was determined. In 'cells with fragmented mitochondria', >50% of the particles displayed a major axis shorter than 1 *μ*m, and in 'cells with tubular mitochondria', >50% of the particles displayed a major axis longer than 1 *μ*m. For the morphometric analysis of mitochondrial particles (Supplementary Figures S1D–F), a 625 *μ*m^2^ large region of interest in the peripheral region of the MEFs was analyzed. Size and perimeter were measured for each mitochondrial particle. To determine mitochondrial shape, the form factor was calculated by using the equation perimerter^2^/4π*area, similarly to previous studies.^26, 27, 28^ Particles smaller than 0.1 *μ*m^2^ were excluded from the analysis.

For Figures 1d and 3g and Supplementary Figure S1B, analyses of mitochondrial morphology was carried out after 30 h of siRNA incubation as described previously and following OH-TAM treatment for 48 h. Cells were seeded in ibidi *μ*-slide eight-well plate (Ibidi, Martinsried, Germany) at a density of 5 × 10^3^ cells/well. Cells were allowed to incubate for 30 min with 200 nM Mitotracker DeepRed, a red fluorescent dye that stains mitochondria in living cells (Invitrogen), following 4% PFA fixation for 20 min. Red fluorescence was excited at 620 nm and emissions were detected through a 690 nm long pass filter. Mitochondrial morphology was separated into three categories as described previously.^56^ Quantification analysis was performed in three independent experiments with at least 300 cells per condition counted without knowledge of treatment conditions.

### DRP1 knockdown

Silencing of DRP1 was performed according to the established protocols.^58^ Briefly, DRP1-siRNA (5′-AAG CAG AAG AAU GGG GUA AAU TT-3′ Eurofins, Luxembourg, Luxembourg) or universal negative control siRNA#1 (Sigma Aldrich) were dissolved separately in Optimem I (Invitrogen). After 5 min of equilibration at RT, each siRNA solution was combined with the respective volume of the Lipofectamine RNAiMAX (Invitrogen) solution, mixed gently and allowed to form siRNA liposomes for 20 min at RT. The transfection mixture was added to the antibiotic-free cell culture medium to a final concentration of 40 nM siRNA and 2.4 *μ*l/ml Lipofectamine. Controls were treated with 100 *μ*l/ml Optimem only.

### Biochemistry

For the generation of whole cell protein lysates, MEFs were washed once in PBS and resuspended in RIPA buffer containing 50 mM Tris-HCl (pH 7.5), 150 mM KCl, 1 mM EDTA, 0.1% SDS, 0.5% sodium deoxycholate and 1% Triton X-100, supplemented with a complete protease inhibitor cocktail (Roche, Basel, Switzerland). When necessary, RIPA buffer was supplemented with the phosphatase inhibitor cocktail PhosSTOP (Roche). After incubation on ice for 30 min, samples were homogenized by sonification and debris was removed by centrifugation (15 min, 10 000 g, 4 °C). Thereafter, protein lysates were mixed with 10 × loading buffer containing 312 mM Tris-HCl (pH 6.8), 10% sodium dodecyl sulfate (SDS), 50% glycerol, 50 mM dithiothreitol and 0.01% bromophenol blue, and separated by SDS-polyacrylamide gel electrophoresis using the BioRad Mini Protean System. After separation, proteins were blotted onto polyvinylidene fluoride membranes (Roti-PVDF; Roth, Karlsruhe, Germany) by using a wet blot apparatus (BioRad). For protein detection, membranes were incubated for 30 min at RT in 5% milk powder in T-TBS containing 150 mM KCl, 20 mM Tris-HCl (pH 7.4), 0.1% Tween-20 (Roth), followed by an incubation step with the primary antibody in 5% milk powder in T-TBS. Dilution of primary antibodies is indicated below. After washing, membranes were incubated for 1 h at RT with the appropriate horseradish peroxidase-conjugated secondary antibody (Pierce, Rockford, IL, USA), diluted 1:1000 in 5% milk powder in T-TBS. Thereafter, membranes were washed again and incubated with chemiluminescence reagents (Western Lightning; PerkinElmer, Rodgau, Germany), according to the manufacturer’s instructions. Chemiluminescence was detected with LI-COR Odyssey FC imaging system (LI-COR Biosciences, Bad Homburg, Germany). To determine the F/G-actin ratio, MEFs were grown to 90% confluence, washed once with pre-heated PBS and harvested in 2 ml PHEM buffer (pH 7.4) containing (in mM) 600 piperazinen,*N*-bis(2-ethanesulfonic acid), 200 HEPES, 100 ethylene glycol tetraacetic acid (EGTA), 20 MgCl_2_ and 10% Triton X-100. The pellet was homogenized using a tight fitting douncer and lysed for 15 min on ice. Thereafter, the samples were centrifuged (20 000 × *g*, 10 min, 4 °C) to separate insoluble from soluble proteins. Eighty percent of the supernatant was mixed with 5 × loading buffer. Residual supernatant was discarded and the pellet was washed with 500 *μ*l PHEM buffer. The pellet was resuspended in the original volume of PHEM buffer and mixed with 5 × loading buffer. Equal volumes of pellet and supernatant were loaded onto a 12% SDS-PAGE and analyzed by western blotting. Quantification of the actin signals in both protein fractions was performed with Quantity One (BioRad), and the ratio of the F-actin to the G-actin signal was calculated. For isolation of mitochondria at least 5 × 10^7^ MEFs are required. MEFs were grown in 10 cm plates until they reached 90% confluence and washed once with pre-heated PBS. Thereafter, MEFs were harvested in 2 ml ice-cold PBS and pelleted by centrifugation (5 min, 500  × *g*, 4 °C). The pellet was washed once in 10 ml ice-cold MSH buffer containing (in mM) 210 mannitol, 70 sucrose, 5 HEPES (pH 7.5) and 1 EDTA (pH 7.5), and resuspended in 4 ml MSH buffer containing complete protease inhibitor cocktail. Disruption of the plasma membrane was achieved by homogenizing the pellet using a tight fitting douncer. To remove unbroken cells, the homogenate was centrifuged (5 min, 900 × *g*, 4 °C). The supernatant was re-centrifuged (15 min, 5500 × *g*, 4 °C) to separate mitochondria from cytosolic proteins. Thereafter, mitochondria were resuspended in 10 ml MSH buffer and centrifugation was repeated twice to ensure purification of mitochondria. Finally, mitochondria were resuspended in MSH buffer at a protein concentration of 80–100 mg/ml.

For investigating DRP1-siRNA efficiency, western blot analysis was performed as described before.^58^ Briefly, 1.2 × 10^5^ cells were seeded into a six-well plate and allowed to incubate with the siRNA transfection mixture for 30 h. At indicated time points, cells were harvested by mild trypsinization with 1 × Trypsin-EDTA, washed once with PBS and lysed in 120 *μ*l protein lysis buffer (pH 7.8, 0.25M d-Mannitol, 0.05 M Trizma base, 1 mM EDTA, 1 mM EGTA, 100 mM DTT, 1% Triton X-100, 1 tablet of Complete mini protease inhibitor cocktail, 1 tablet of phosphatase inhibitor, both from Roche Diagnostics). This step was performed on ice. The harvested cells were put in liquid N_2_ for 3 min and thawed on ice. Afterwards, cell lysate was centrifuged at 10 000 r.p.m. for 10 min at 4 °C to eliminate insoluble fragments. The supernatant was used for western blot analysis. The Pierce BCA Kit (Perbio Science, Bonn, Germany) was used for quantifying the whole protein amount. For western blot analysis, 40 *μ*g of protein were loaded on a 10% SDS gel and blotted onto a PVDF membrane at 40 mA for 21 h. Incubation with primary antibody was performed overnight at 4 °C.

### Mitochondrial membrane potential

To quantify membrane potential, cells were stained with the potentiometric fluorescent tetramethylrhodamine ethyl ester (TMRE) dye (MitoPT TMRE kit; Immunochemistry Technologies, Bloomington, MN, USA). Cells were seeded in a 24-well plate at a density of 4–11 × 10^3^ cells/well and incubated with OH-TAM for 72 h. Cells were collected by trypsinization and incubated for 30 min at 37 °C with TMRE dye. Afterwards, cells were washed with PBS and resuspended in 1 × assay buffer. Carbonyl cyanide *m*-chlorophenyl hydrazone (CCCP) protonophore was applied at a concentration of 50 *μ*M for 30 min before TMRE staining. This was used as a positive control to induce a complete loss of mitochondrial membrane potential. Fluorescence was measured by Guava EasyCyte Flow Cytometer (Merck Millipore, Billerica, MA, USA) at the excitation wavelength of 488 nm and emission was measured at 680 nm. At least 3000 cells per condition were evaluated in three independent experiments with *n=3* per sample.

### Mitochondrial production of ROS

To quantify production of mitochondrial-derived ROS, MEF cells were seeded in a 24-well plate at a density of 4–11 × 10^3^ cells/well. After 72 h of OH-TAM treatment cells were stained with the live-cell permeable MitoSOX Red dye (Life Technologies, Carlsbad, CA, USA) following the manufacturers’ protocol. Afterwards, cells were collected, centrifuged and washed with PBS. Changes in red fluorescence were analyzed with Guava EasyCyte Flow Cytometer (Merck Millipore) at an excitation wavelength of 488 nm and emission was recorded at 680 nm. Data were collected from 3000 cells from three wells per condition in three independent experiments.

### ATP measurements

For luminescence-based ATP measurements, MEF were seeded at a density of 1.9 × 10^3^ cells/well in a white 96-well plate (Greiner, Kremsmünster, Austria). ATP levels were analyzed by luminescence detection (FluoStar; BMG Labtech, Ortenberg, Germany) according to the manufacturer’s protocol using the ViaLight plus kit (Lonza, Basel, Switzerland) after 48 h and 72 h of OH-TAM incubation. Data are representative for three independent experiments (*n*=7 per treatment condition).

### Cellular OCR and ECAR

For detection of changes in the mitochondrial respiration, MEF cells were seeded in a XF96-well microplate (Seahorse Bioscience, Santa Clara, CA, USA) with a density of 1.9 × 10^3^ cells/well. Cells were incubated with OH-TAM for 24, 48 or 72 h and OCR/ECAR was measured as previously described.^68^ Briefly, 1 h before the measurement started the growth medium was replaced by 180 *μ*l of assay medium (25 mM glucose, 2 mM glutamine, 1 mM pyruvate, pH 7.35) and incubated at 37 °C. Three baseline measurements were recorded before injecting different compounds. Port A contains oligomycin at a final concentration of 3 *μ*M, Port B contains 1 *μ*M carbonyl cyanide-4-(trifluoromethoxy)phenylhydrazone, Port C contains a combination of 1 *μ*M antimycin A and 100 nM rotenone and Port D contains 50 mM 2-desoxyglucose. Three measurements were performed after injection of each compound. Data are representative for three independent experiments with an *n=*8 per treatment condition.

### Primary antibodies

mouse anti-cytochrome *c* (2 h, RT, 1:1000, clone 7HB8.2C12; BD Pharmingen, Franklin Lakes, NJ, USA), rabbit anti-TOM20 (1:200; Santa Cruz, Dallas, TX, USA), mouse *β*-tubulin (2 h, RT, 1:1000 in immunocytochemistry (IHC) and in western blots, clone TUB2.1, Sigma Aldrich), mouse anti-DRP1 (2 h, RT, 1:1000 in IHC and western blot; BD Bioscience, Franklin Lakes, NJ, USA) for Figure 3a/c and Supplementary Figures S3A–C, mouse anti-DRP1 (2 h, RT, 1:800; BD Bioscience) for Figure 3d, rabbit anti-p-DRP1 (1:500; Cell Signalling, Danvers, MA, USA), mouse anti-actin (1:1000; MP Biomedicals, Santa Ana, CA, USA), mouse anti-KDEL (o/n, 4 °C, 1:500; Abcam, Cambridge, UK), mouse anti-PDI (o/n, 4 °C, 1:1000; Enzo Life Sciences, Lörrach, Germany), rabbit anti-GM130 (o/n, 4 °C, 1:250; Abcam), mouse anti-giantin (o/n, 4 °C, 1:1000; Enzo Life Sciences). Generation of rabbit anti-COXII (1:1000) has been described before.^22^ Alexa Fluor 488-conjugated phalloidin (1:500; Invitrogen) was used to visualize F-actin.

### Statistics

If not otherwise stated, mean values and standard error of the means (S.E.M.) were shown in the graphs. In some graphs of Supplementary Figure S2, standard deviation was shown instead of S.E.M. For statistical analyses, Student’s *t*-test was performed when comparing with data sets with normal distribution. When comparing various experimental conditions with small *n*, the ANOVA Scheffé test was performed. During all image analyses, the experimenter was blind to the genotype or treatment condition.

## Publisher’s Note:

Springer Nature remains neutral with regard to jurisdictional claims in published maps and institutional affiliations.
