# Supplementary figures and images for: Cofilin1-dependent actin dynamics control DRP1-mediated mitochondrial fission
Source: Cell Death Dis. 2017 Oct 5;8(10):e3063–. doi: 10.1038/cddis.2017.448 (PMC5680571; doi:10.1038/cddis.2017.448)

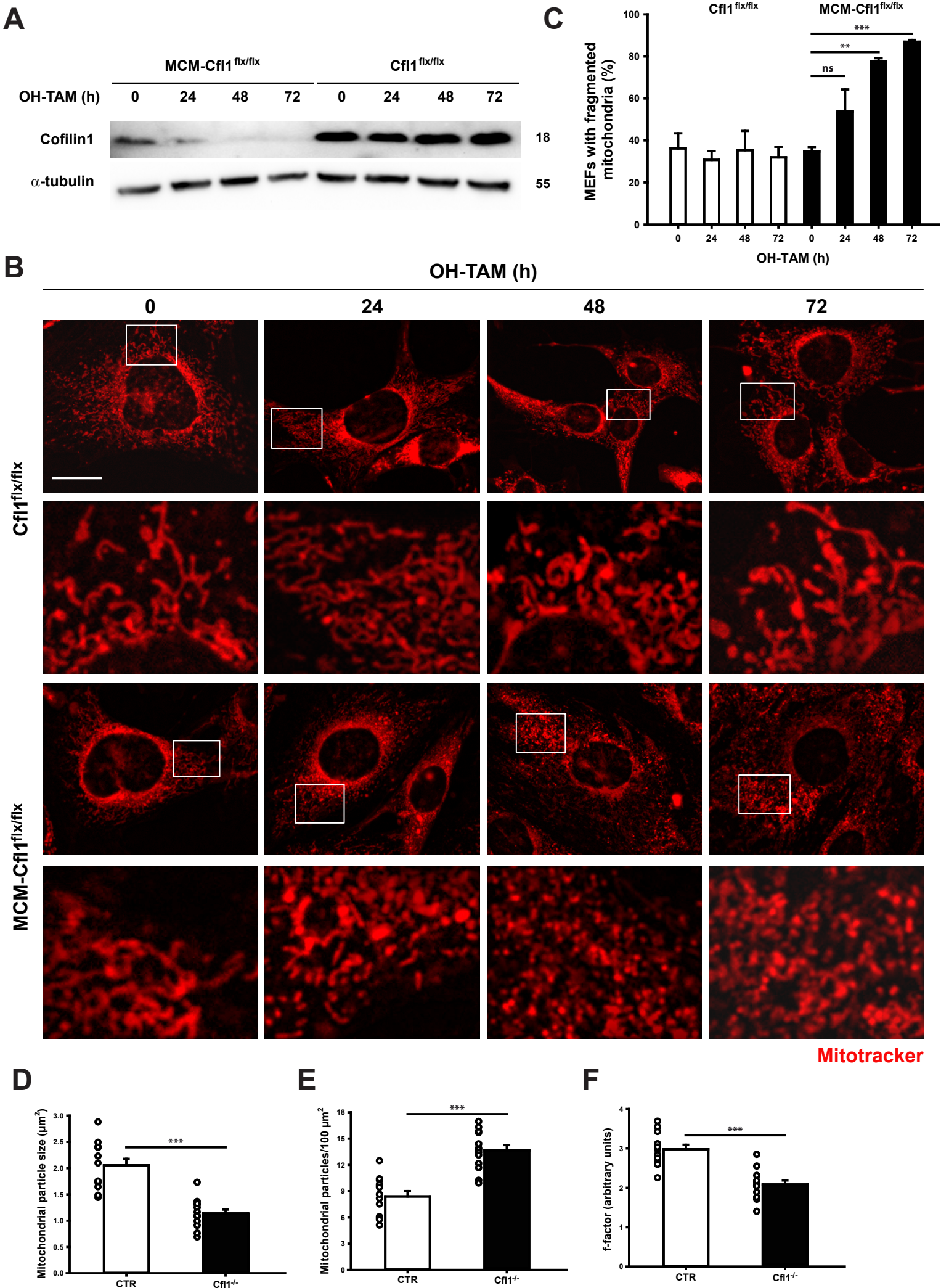

Figure S1

Supplement: Supplementary Figure 1 [file cddis2017448x1.pdf]

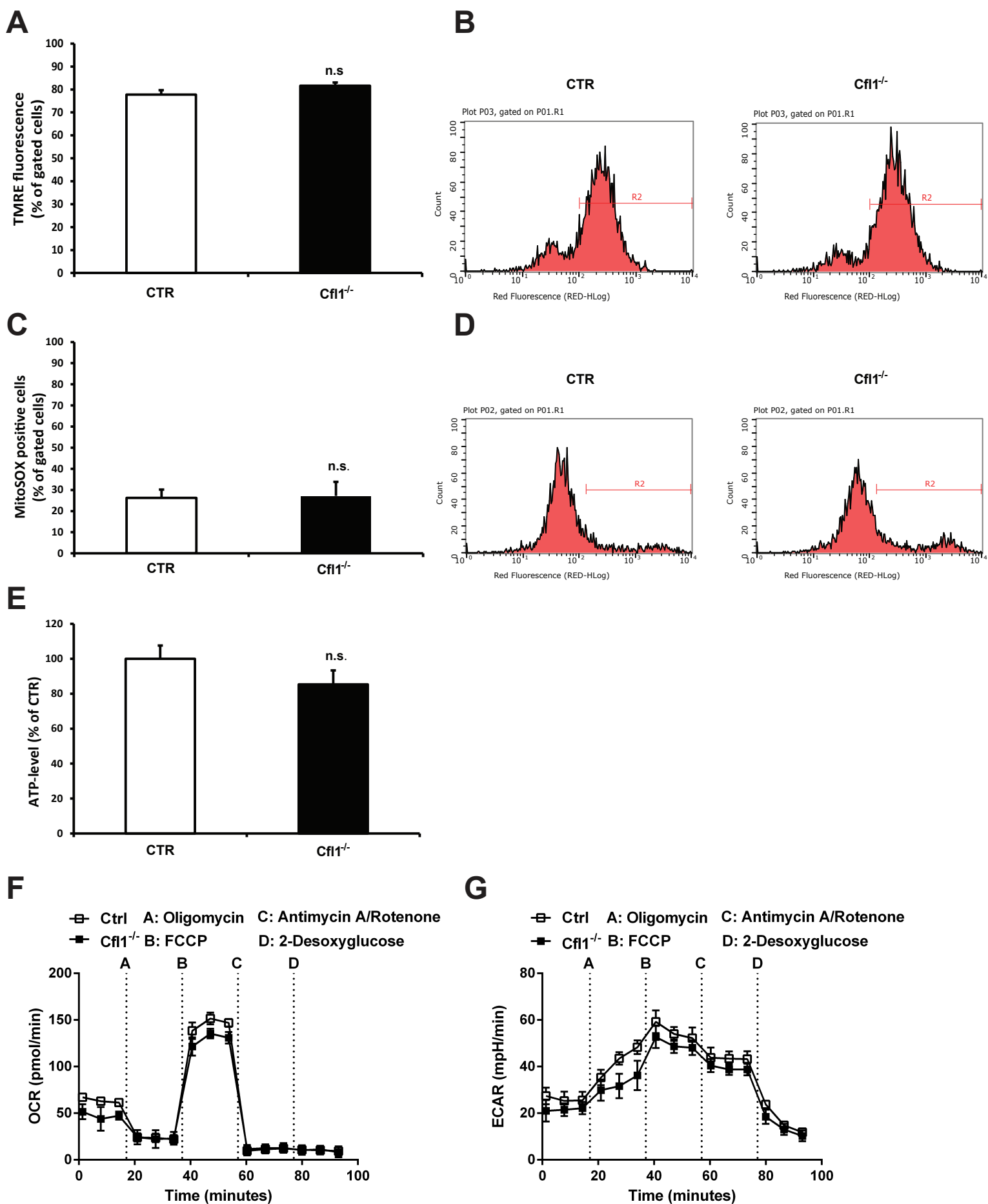

Figure S2

Supplement: Supplementary Figure 2 [file cddis2017448x2.pdf]

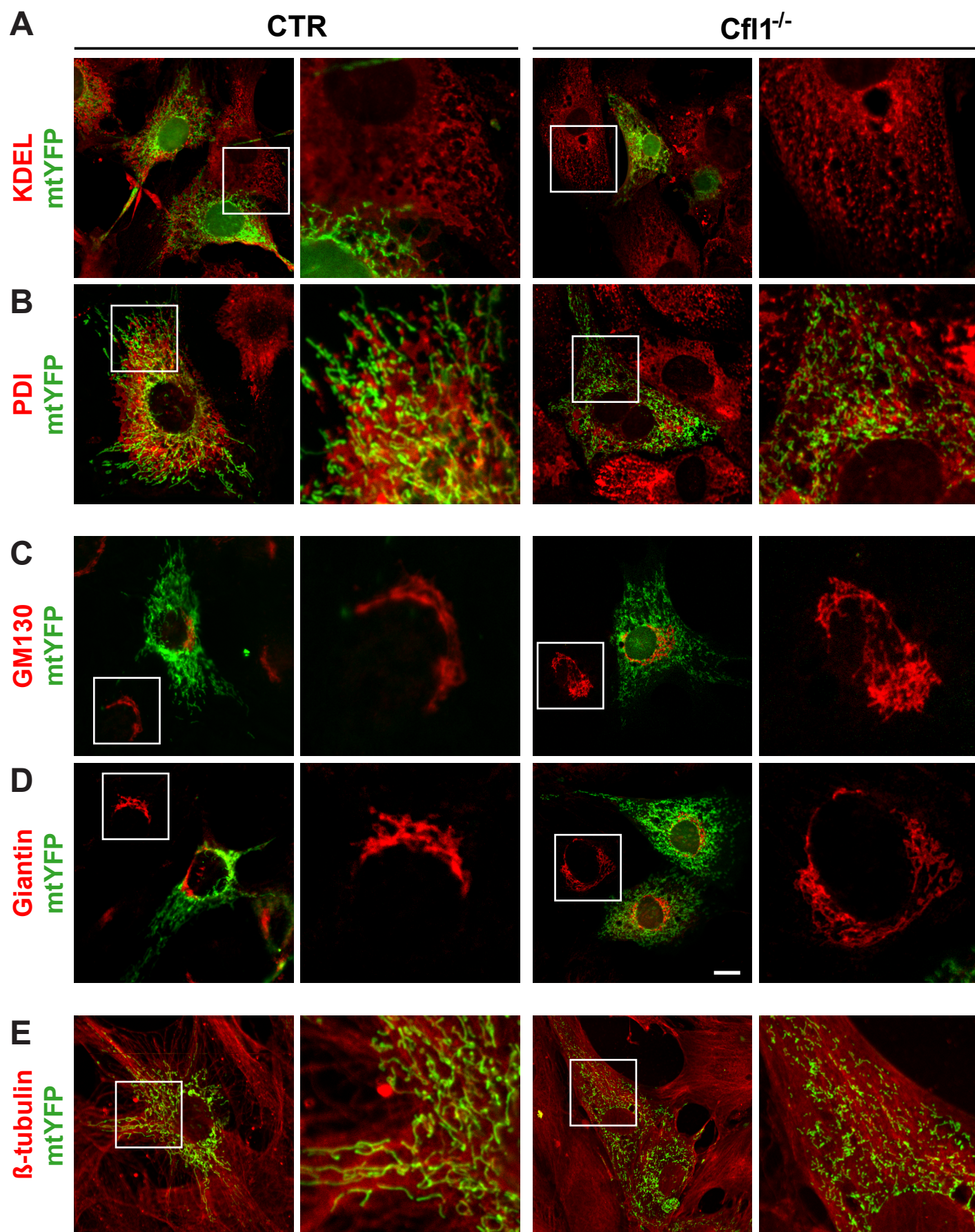

Figure S3

Supplement: Supplementary Figure 3 [file cddis2017448x3.pdf]

**A**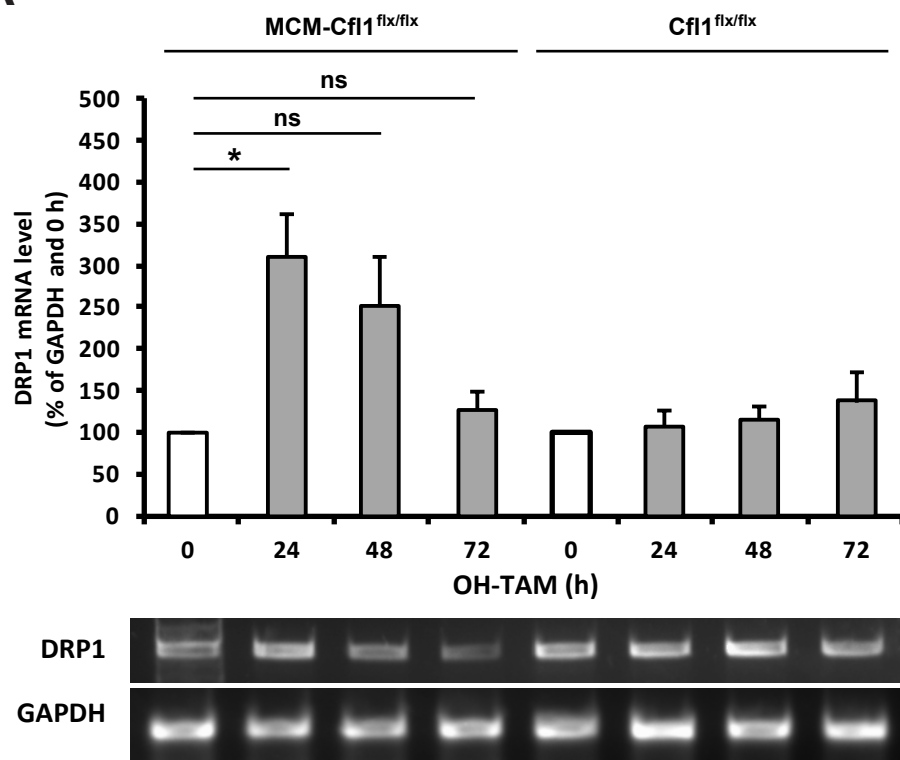**B**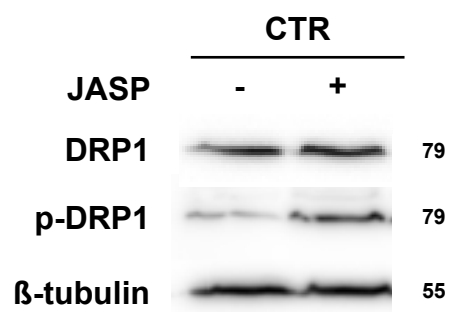**C**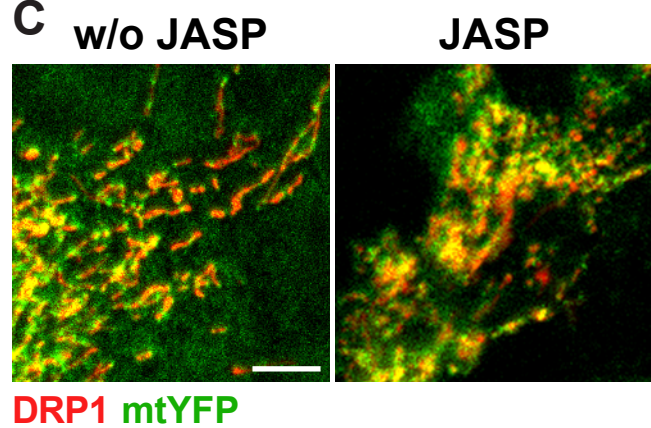**D**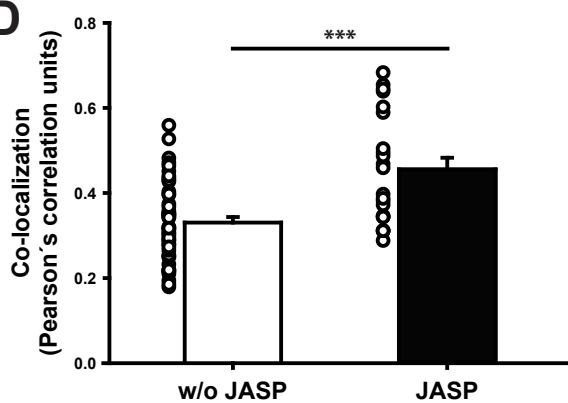**Figure S4**

Supplement: Supplementary Figure 4 [file cddis2017448x4.pdf]
