# Supplementary material for: Cofilin1-dependent actin dynamics control DRP1-mediated mitochondrial fission
Source: Cell Death Dis. 2017 Oct 5;8(10):e3063–. doi: 10.1038/cddis.2017.448 (PMC5680571; doi:10.1038/cddis.2017.448)
Supplement: Supplementary Figure Legends [file cddis2017448x5.docx]

**Cofilin1-dependent actin dynamics control DRP1-mediated mitochondrial fission**

**SUPPLEMENTARY FIGURE LEGENDS**

**Figure S1. Time course of cofilin1 depletion and mitochondrial fragmentation in OH-TAM-treated MCM-Cfl1^flx/flx^-MEFs. (A)** Western blot demonstrating absence of cofilin1 from MCM-Cfl1^flx/flx^-MEFs after 48 h of OH-TAM treatment. Conversely, OH-TAM did not deplete cofilin1 in Cfl1^flx/flx^-MEFs that did not express MCM. α-tubulin served as a loading control. **(B)** Representative micrographs of Mitotracker-stained, OH-TAM-treated Cfl1^flx/flx^-MEFs that either or not expressed MCM. White boxes indicate areas shown at high magnification. Scale bar: 10 µm. **(C)** Quantification of mitochondrial fragmentation in OH-TAM treated Cfl1^flx/flx^-MEFs that either or not expressed MCM. Shown are relative numbers of MEFs with fragmented mitochondria. We confirmed mitochondrial fragmentation in Cfl1^-/-^-MEFs by a detailed morphometric analysis of randomly chosen MEFs. This analysis revealed **(D)** a reduction in mitochondrial particle size (CTR: 2.05±0.12 µm^2^, n=13 micrographs from 13 cells, 8,125 µm^2^ analyzed area; Cfl1^-/-^: 1.14±0.08µm^2^, n=13/13/8,125 µm^2^; P<0.001), **(E)** an increased mitochondrial particle density (CTR: 8.39±0.61 organelles/100 µm^2^; Cfl1^-/-^: 13.65±0.61 organelles/100 µm^2^; P<0.001), and **(F)** a reduced form-factor (f-factor) in Cfl1^-/-^-MEFs (CTR: 2.98±0.11; Cfl1^-/-^: 2.08±0.10; P<0.001). Columns and error bars in C-F: MV+SEM. Open circles in D-F: values of individual MEFs. **: P<0.01, ***: P<0.01, ns: not significant.

**Figure S2. Normal mitochondrial functions in Cfl1^-/-^-MEFs. (A)** Tetramethyl rhodamine ethyl ester (TMRE) fluorescence quantified by FACS analysis revealed that mitochondrial membrane potential (MMP) was not altered in Cfl1^-/-^-MEFs. **(B)** Representative FACS plots showing that mitochondrial fragmentation upon cofilin1 inactivation had no impact on MMP. **(C)** Mitochondrial production of reactive oxygen species (ROS) detected by MitoSOX staining and subsequent FACS analysis was unchanged in Cfl1^-/-^-MEFs. Columns and error bars in A, C: MV±SD of three independent experiments. **(D)** Representative FACS plots showing that mitochondrial ROS level were not altered upon cofilin1 depletion. **(E)** ATP production was unchanged in Cfl1^-/-^-MEFs. Columns and error bars: MV±SD of seven independent experiments. **(F)** Measurement of oxygen consumption rate (OCR) revealed no differences between CTR-MEFs and Cfl1^-/-^-MEFs in basal or FCCP-induced maximal respiration **(G)** Likewise, the extracellular acidification rate (EAR) was unchanged in Cfl1^-/-^-MEFs implicating that mitochondria showed no shift to glycolysis upon cofilin1 depletion. Squares and error bars in F, G: MV±SD of eight independent experiments.

**Figure S3. Absence of obvious morphological changes of the endoplasmic reticulum, the Golgi apparatus, and the microtubule cytoskeleton in Cfl1^-/-^ MEFs.** Antibodies against various proteins or sorting motifs were exploited to visualize the **(A-B)** endoplasmatic reticulum (KDEL, PDI), **(C-D)** the Golgi apparatus (GM130, giantin), and **(E)** the microtubule cytoskeleton (β-tubulin) in mtYFP-expressing MEFs. None of these structures appeared obviously altered in Cfl1^-/-^-MEFs. White boxes in the left micrographs indicate areas magnified in right micrographs. Scale bar in D: 50 µm.

**Figure S4: Unchanged DRP1 mRNA in Cfl1^-/-^-MEFs and increased mitochondrial DRP1 localization in CTR-MEFs upon pharmacological actin stabilization. (A)** Representative gels and statistical analysis of DRP1 mRNA levels in untreated (0 h) and OH-TAM-treated Cfl1^flx/flx^-MEFs that either or not express MCM. GAPDH served as a loading control and was used for normalization. Columns and error bars: MV±SEM of five independent experiments. ns: not significant, *: P<0.05. **(B)** Representative Western blot analysis revealed unaltered DRP1 levels, but increased p-DRP1 (S616) levels upon JASP treatment in CTR-MEFs. β-tubulin was used as a loading control. **(C)** DRP1 immunoreactivity (red) in untreated (w/o JASP) and JASP-treated, mtYFP-expressing CTR-MEFs. Scale bar: 10 µm. **(D)** JASP treatment increased the Pearson´s correlation of mtYFP and DRP1 in CTR-MEFs (w/o JASP: 0.33±0.01, n=49; JASP: 0.45±0.03, n=22, P<0.001). Columns and error bars: MV+SEM. Open circles: values of independent experiments. ***: P<0.001.
